# Normalization of elevated cardiac, kidney, and hemolysis plasma markers within 48 h in Mexican Tarahumara runners following a 78 km race at moderate altitude

**DOI:** 10.1002/ajhb.22607

**Published:** 2014-08-22

**Authors:** Dirk L Christensen, Diana Espino, Rocío Infante-Ramírez, Soren Brage, Dijana Terzic, Jens P Goetze, Jesper Kjaergaard

**Affiliations:** 1 Unit of International Health, University of CopenhagenCopenhagen, Denmark; 2 Faculty of Physical Education and Sport Sciences, Autonomous University of ChihuahuaChihuahua, México; 3 Faculty of Chemical Sciences, Autonomous University of ChihuahuaChihuahua, México; 4 MRC—Epidemiology Unit, Addenbrooke's Hospital, University of CambridgeUnited Kingdom; 5 Department of Clinical Biochemistry, Centre of Diagnostics, Copenhagen University HospitalRigshospitalet, Denmark; 6 Department of Cardiology, Heart Centre, Copenhagen University HospitalRigshospitalet, Denmark

## Abstract

**Objectives:**

The aim of this study was to examine to what extent extreme endurance exercise results in changes of plasma markers associated with cardiac and renal damage, as well as hemolysis in male, Mexican Tarahumara runners.

**Methods:**

Ten Tarahumara runners (mean (sd) age of 38 (12) years) participated in a 78 km race in Chihuahua, Mexico at 2,400 m above sea level. Cardiac, kidney, and hematology plasma markers were measured pre-race and <5 min, 1 h, 3 h, 6 h, 24 h, and 48 h post-race. Anthropometry, blood pressure, pulse rate, electrocardiography, HbA1c, hemoglobin and VO_2max_ (estimated from heart rate following step test) were assessed pre-race, while physical activity energy expenditure and intensity were estimated during the race, and oxygen partial pressure saturation (SpO_2_) <30 min post-race.

**Results:**

Estimated mean VO_2max_ was 48 (9) mLO_2_ min^−1^ kg^−1^ and relative intensity during the race was 68 (11)%VO_2_max. Mean SpO_2_ was 92 (3)% <30 min post-race. Plasma concentrations of especially total creatine kinase, creatine kinase-MB isoform, and haptoglobin changed significantly from pre-race values (*P <* 0.001) up to 24 h post-race, but had returned to pre-race values after 48 h. The plasma concentrations of mid-regional proatrial natiuretic peptide and copeptin returned to pre-race concentrations after 1 and 6 h, respectively.

**Conclusions:**

Altered cardiac, renal, and hemolysis plasma markers were normalized after 48 h following 78 km of running, suggesting that the impact of exercise-induced cardiac and kidney damage as well as hemolysis in the Mexican Tarahumara is low. Am. J. Hum. Biol. 26:836–843, 2014. © 2014 The Authors American Journal of Human Biology Published by Wiley Periodicals, Inc.

Cardiovascular physiology and biochemistry in endurance exercise has been the subject of increasing interest over the last couple of decades. Concerns have been raised that regular participation in ultra-endurance sports may result in exercise-induced cardiomyopathy, as several studies have documented changes in surrogate measures in plasma associated with cardiac damage (Lippi et al., 2012b; Roth et al., 2007a; Scott et al., 2009). Among these markers are the cardiac troponins and natriuretic peptides (Scott et al., 2009) which are associated with myocardial hypoxia and myocardial wall-stretch, respectively (Hammerer-Lercher et al., 2001; Thygesen et al., 2007). More recently, other plasma markers that may be useful in assessing cardiac damage have been identified. One example is copeptin, which is a stable peptide derived from the precursor of vasopressin (Bahrmann et al., 2013). Together, these plasma markers may indicate the degree of exercise-induced cardiomyopathy and—by repeated post-exercise measures—further indicate to what extent changes in plasma concentrations are transient.

The majority of studies on markers in plasma and exercise-induced cardiomyopathy have been performed in apparently healthy individuals (see for example (Lippi et al., 2012b)). To the best of our knowledge, no studies have included individuals of Native American origin. Among Native Americans in North America, the Mexican Tarahumara are among the few who still maintain the once widespread tradition of ultra-distance running (Rascón and Batista, 1994). As they are exposed to regular long-distance running from childhood, they may be less susceptible to exercise-induced cardiomyopathy compared to athletes who have taken up endurance training in adulthood which is common in the so-called “Western” culture (La Gerche et al., 2012).

The aim of the study was to (a) establish the effects of ultra-marathon running primarily on plasma markers of cardiac damage, and (b) to evaluate the increase in plasma markers as surrogate measures of kidney injury and hemolysis up to 48 h post-race in male Tarahumara runners accustomed to life-long endurance exercise.

## RESEARCH DESIGN AND METHODS

### Study site and population

Ten adult men from the village of Choguita, Chihuahua, volunteered to participate in a 78 km running race organized for the purpose of the study. The number of included participants was based on availability of subjects at the time of the study. The event took place in November 2011, and began at 5:55 a.m. outside a small hospital, Centro Avanzado de Atencion Primaria a la Salud (CAAPS) in the town of Guachochi at an altitude of ∼2,400 m; the participants had to cover a flat loop of 26 km three times on a dirt road, and they were encouraged to cover the 78 km as fast as possible. All participants wore their traditional shirts and loincloth as well as homemade sandals, except for one participant who wore cushioned running shoes. Information on several biological parameters were collected prior to as well as post-race (details are given below). All participants gave written and oral informed consent. In case of illiteracy, the consent form was signed with a thumb print. Ethical approval was given by the Ethical Committee of Science in Chihuahua, Mexico (no. 0003925).

### Anthropometry

Height and weight were measured to the nearest 0.1 cm and 0.1 kg, respectively with the participant wearing minimal clothing, and body mass index (weight in kg/height in m^2^) was derived.

### Blood pressure and pulse rate

Systolic and diastolic blood pressures as well as resting pulse rate were measured with a full-automatic monitor (Omron M8 RC, Kyoto, Japan). Blood pressure was measured three times on the right upper arm with the participant seated and following 15 min of rest. Mean blood pressure was calculated from the last two measurements; the first measurement was disregarded in order to minimize influence of “white coat syndrome.” Hypertension was defined as systolic blood pressure ≥140 mm Hg and/or diastolic blood pressure ≥90 mm Hg (Whitworth 2003).

### Electrocardiography

A standard 12-lead electrocardiography (ECG) was performed using a Medi Trace, ver. 5.2 (Medi Core, Mexico City). The ECG was read by an experienced cardiologist (J.K.), blinded to participant demographic and race data.

### Cardiac, kidney, and hematology plasma markers

Ten ml blood was collected in lithium heparin-containing BD-Vacutainer® tubes at each time point, i.e. at pre-race (baseline), <5 min post-race, 1 h post-race, 3 h post-race, 6 h post-race, 24 h post-race, and 48 h post-race using an intravenous catheter. Blood was centrifuged and the plasma was stored in cryogenic tubes at −80°C, and transported with dry ice to Copenhagen University Hospital, Denmark for analyses.

Heparinized plasma was analyzed on an automated platform for high-sensitivity cardiac troponin T (hs-cTnT), creatine kinase (CK), and CK isoform myocardial band (MB), creatinine, high sensitivity C-reactive protein (hs-CRP), iron, haptoglobin, lactate dehydrogenase (LDH) (Roche Modular E170), all analyses were accredited by a National organ (DANAK) and constantly subject of external validation programs. For mid-regional proatrial natiuretic peptide (MR-proANP) and copeptin ultra-sensitive (copeptin-us), we used the Kryptor Compact Plus platform (Thermo-Fisher, Germany); the validation of the two analyses has recently been reported by us (Hunter et al., 2011;Terzic et al., 2012).

### HbA1c and hemoglobin

Glycosylated hemoglobin (HbA_1c_) and hemoglobin levels were determined by using 10 µL of capillary blood. HbA1c was analyzed using an automated boronate affinity assay which minimizes interference from other hemoglobin variants and derivates analyzed by an Afinion AS100 Analyzer (Axis Shield PoC, Oslo, Norway). Hemoglobin was analyzed by a modified azide-methemoglobin reaction (Vanzetti 1966) using a Hb 201+ device (HemoCue AB, Ängelholm, Sweden). Anemia was defined as blood hemoglobin below 13 mg/dL.

### VO_2max_ and race energy expenditure

The assessment of cardio-respiratory fitness (VO_2max_) and race energy expenditure was carried out using a combined uniaxial accelerometer and heart rate sensor (Actiheart, CamNtech Ltd, Cambridge, UK). Two days prior to the staged race, the monitor was applied to the chest on two ECG electrodes (Unomedical A/S, Birkerød, Denmark); a medial electrode placed at the lower part of the sternum, and a lateral electrode placed on the same horizontal level and as laterally as possible without stretching the wire below the major pectoral muscle (Brage et al., 2006).

Prior to the race, each participant was asked to perform an 8-min step test, stepping up and down a 21.5 cm high step (Reebok, Lancaster, UK). The stepping frequency was 15 step cycles (body lifts) per minute in the first minute, after which it increased linearly up to 33 steps per minute at the end. The test was followed by 2 min of sitting recovery. Heart rate recovery was measured for at least 90 sec after the step test, regressed against recovery time, and expressed as the HR above sleep at 1 min post stepping (Brage et al., 2007). The individual heart rate response to the step test was used to calibrate HR to protocol-estimated physiological intensity, and also to obtain an estimate of VO_2max_ (expressed as mLO_2_ min^−1^·kg^−1^) by extrapolation of the sub-maximal relationship to age-predicted maximal heart rate (Tanaka et al., 2001) and adding an estimate of resting metabolic rate (Henry, 2005).

Following the step test procedure, the combined sensor was downloaded and re-initialized for monitoring the running race, collecting data every 15 seconds. All long-term heart rate data were pre-processed using a robust Gaussian Process Regression method (Stegle et al., 2008), and sleeping heart rate as well as average heart rate above sleep and body movement (accelerometry) during the race were derived. Physical activity intensity was estimated by branched equation modeling (Brage et al., 2004) to combine step-test calibrated heart rate information with the accelerometry-based estimate of intensity (Brage et al., 2007).

### Oxygen saturation

Post-race (<30 min) oxygen partial pressure saturation (SpO_2_) was measured transcutaneously using a portable bedside monitor with an accuracy of ±3% (Philips Medical Systems, SureSigns VM8, Eindhoven, The Netherlands).

### Statistics

All background and descriptive results are given as mean (SD) or median (IQR) if data were skewed. Within subject variation of plasma biomarkers, with age as explanatory variable, using the baseline values as the references was done by repeated measurements (mean (95% CI)). Linear regression analysis was used to test for associations between exposure and outcome variables as well as between different outcome variables. In the case of participants having plasma values below or above the detection limit for a plasma marker, data were presented as median (IQR). Estimated VO_2_max data was missing for one participant, in which case imputation was used on the basis of age and race time (*r*^2^ = 0.75). *P <* 0.05 was considered statistically significant. All analyses were done with the Stata 12.2 Intercooled version (Stata Corp, College Station, TX).

## RESULTS

**Figure 1:**
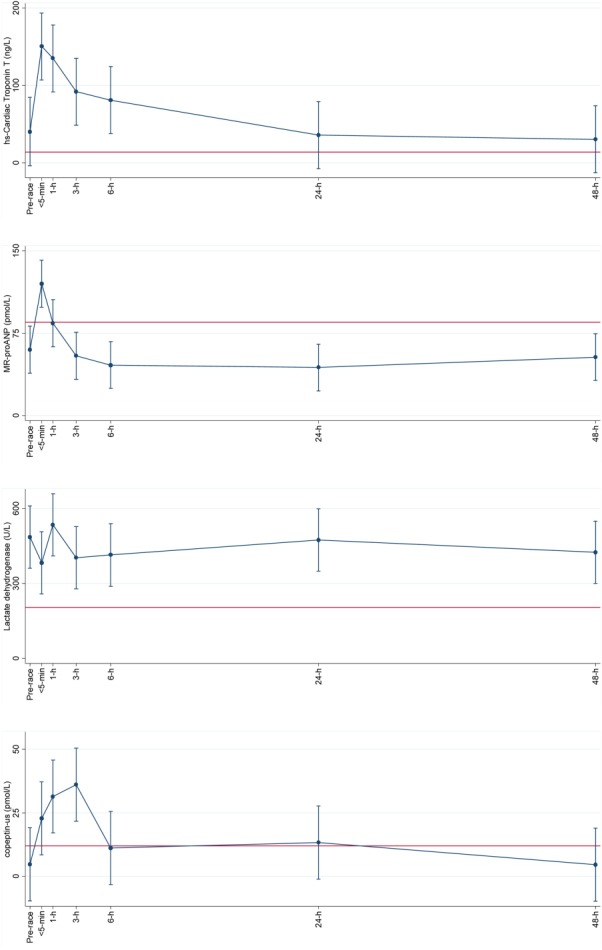
Within subject variation of cardiac plasma markers hs-cardiac TnT (ng/L), MR-proANP (pmol/L), lactate dehydrogenase (U/L), and cardiac/kidney plasma marker copeptin-us (pmol/L) with age as explanatory variable, using the baseline values as the references was done by repeated measurements (mean (95% CI)) at time points <5 min, 1 h, 3 h, 6 h, 24 h, and 48 h after running 78 km. Straight line shows upper normal limit for each plasma marker.

**Figure 2:**
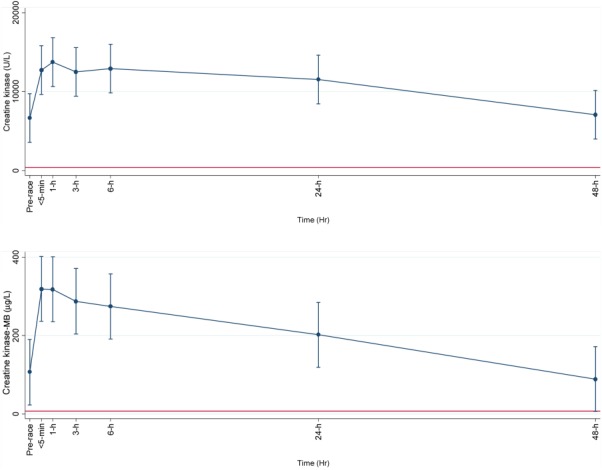
Within subject variation of skeletal muscle and skeletal/cardiac muscle, respectively plasma biomarkers creatine kinase (U/L) and creatine kinase-MB (µmol/L), with age as explanatory variable, using the baseline values as the references was done by repeated measurements (mean (95% CI)) at time points <5 min, 1 h, 3 h, 6 h, 24 h, and 48 h after running 78 km. Straight line shows upper normal limit for each plasma marker.

**Figure 3:**
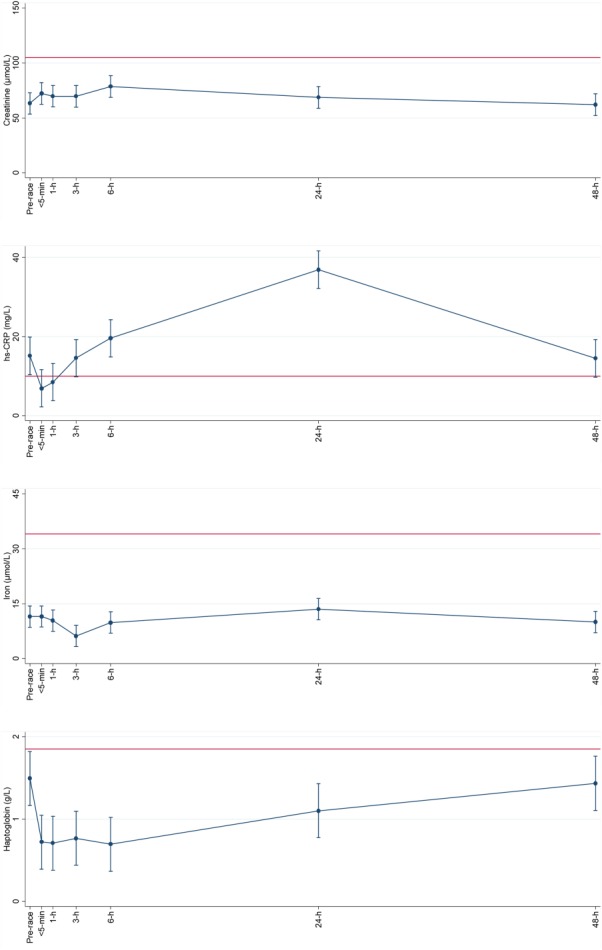
Within subject variation of general, kidney and hemolysis plasma biomarkers hs-CRP (mg/L), creatinine (µmol/L), iron (µmol/L), and haptoglobin (g/L) with age as explanatory variable, using the baseline values as the references was done by repeated measurements (mean (95% CI)) at time points <5 min, 1 h, 3 h, 6 h, 24 h, and 48 h after running 78 km. Straight line shows upper normal limit for each plasma marker.

Mean age of the participants was 38 (±12) years. One had hypertension and two were anemic. Background characteristics are presented in Table1. Estimated VO_2max_ was 48 (9) mL O_2_ min^−1^ kg^−1^. The average activity intensity during the race was 584 (184) J min^−1^ kg^−1^ (about 9.2 METs), or approximately 68 (11)% of estimated VO_2_max. Estimated VO_2max_, heart rate and race-related data are presented in Table2. Measurement of SpO_2_ was done in six subjects who had a mean of 92 (3)% (range: 88–97%) <30 min post-race. Moderate (from 88 to<93%) and mild (from 93 to <95%) exercise-induced hypoxemia was found in four and one subjects, respectively according to the definitions of (Dempsey and Wagner, 1999). The participants had formerly participated in races ranging between 50 and 160 km not including their traditional ultra-distance kick-ball race. For the plasma markers, three participants were below the detection limit for haptoglobin of 0.2 g/L, two were above the upper reference limit of 22,000 U/L for total plasma CK, and two were above the upper detection value for plasma CK-MB of 600 µg/L. Plasma results using the pre-race value as reference showed the most profound differences when adjusted for age. Plasma concentrations of total CK, CK-MB, and haptoglobin showed the most marked changes compared to the pre-race values (*P <* 0.001), while plasma LDH did not show any significant decrease. All concentrations of the ten plasma markers had returned to pre-race levels after 48 h (see Figs. 1–3). A CK-MB: total CK ratio was calculated, and all values except for the post-48 h value were significantly increased compared to the pre-race value (*P <* 0.001). Mean CK-MB: total CK ratio went from 1.4% up to 2.7% <5-min post-race and then gradually decreased towards baseline. The association between total CK and LDH was statistically significant (*P <* 0.001). There were no significant associations between estimated VO_2max_ and post-exercise cTnT (*P =* 0.12) or duration of race time and post-exercise cTnT (*P =* 0.38).

**Table 1:** Background characteristics of Tarahumara runners (n = 10)

Variable	Mean(SD)	Range
Age (yrs)	38 (12)	17–63
Height (cm)	162.8 (3.7)	158.0–169.8
Weight (kg)	60.2 (5.9)	47.8–66.6
BMI (kg/m^2^)	22.7 (1.8)	18.7–24.3
Systolic blood pressure (mm Hg)^a^^b^	122 (14)	103–154
Diastolic blood pressure (mm Hg)^a^	73 (10)	59–88
HbA_1c_ (%)	5.5 (0.2)	5.3–5.8
Hemoglobin (g/dL)^c^	14.8 (1.8)	12.4–17.4

a Blood pressure measured with subject in sitting position ≥15 min of rest.

b One subject had hypertension.

c Two male subjects had anemia (hemoglobin<13.0 g/dL).

**Table 2:** Estimated VO_2_max, heart rate measures, and running intensity of Tarahumara runners (n = 10)

Variable	Mean(SD)/median(IQR)	Range
Estimated VO_2_max (mLO_2_ min^−1^ kg^−1^)^a^	48 (9)	33.9–61.3
Estimated maximal heart rate^b^ (bts min^−1^)	181 (9)	164–196
Sleeping heart rate (bts min^−1^)	59 (9)	43–75
Post-race heart rate (bts min^−1^)	88 (9)	75–101
Race time (h and min)^c^	8.42 (1.52)	6.43–11.41
Absolute intensity (km h^−1^)^c^	9.0 (7.4;11.0)	6.8–12.1
Physiological intensity (J min^−1^ kg^−1^)	584 (184)	384–905
Relative intensity during race (%)	68 (11)	45–84

a One participant did not have a valid step test, and thus his VO_2_max was estimated through imputation.

b Estimated maximal heart rate according to Tanaka equation (Tanaka et al., 2001).

c Data are presented as median (IQR 25;75).

## DISCUSSION

This study shows that total plasma CK and CK-MB increase dramatically in male Tarahumara following a 78 km endurance race. Total plasma CK as well as plasma CK-MB had doubled 24 h post-race, but both markers were back to baseline values 48 h after the race. Together with a significant tripling of hs-cTnT up to 1 h post-race and doubling up to 6 h post-race, this may indicate hypoxia-induced myocardial damage due to prolonged endurance exercise. Several studies have shown a significant increase in similar cardiac plasma markers following ultradistance competition (Middleton et al., 2008;Roth et al., 2007;Scott et al., 2009). Interestingly, Trivax et al. (2010) reported a threefold and fourfold increase in CK and CK-MB, respectively, immediately after a marathon race with a further increase 24 h post-race. This significant difference compared to the current results could be explained by higher work intensity in the marathon runners (∼10 km h^−1^ vs. 9 km h^−1^).

The elevated post-exercise levels of total CK and its isoform CK-MB indicate some degree of cardiac damage. A CK-MB to total CK ratio of above 5 to 6% has been suggested as a clinical cut-off for acute myocardial infarction and thus an exercise-induced CK-MB to total CK ratio above this threshold apparently indicates myocardiac damage (Cummins et al., 1987). Even though we found a mean CK-MB to total CK ratio <3%, which has been suggested to reflect exercise-stimulated skeletal muscle degradation rather than isolated release of CK-MB (Lucia et al., 1999), we cannot rule out the possibility that the increased fraction of CK-MB may still indicate some degree of myocardial damage.

The most commonly used plasma marker to assess exercise-induced cardiomyopathy are the different forms of cardiac troponins as they are highly specific markers of myocardial cell damage (Collinson et al., 2001). All participants had baseline values above the diagnostic threshold (3 µg/L) for hs-cTnT and post-race values increased by up to 12-fold immediately after the race at the individual level (approximately fourfold at group level). Even though this could lead one to speculate that some of the participants had undetected cardiomyopathy, it seems unlikely since the time course pattern of cTnT as well as CK-MB was similar in all ten participants, only at different absolute levels. Furthermore, none of the participants reported current or previous cardiomyopathy, and all participants had normal resting ECGs.

It has been suggested that differences in, among others, estimated VO_2max_ levels of participants or duration of exercise, i.e. intensity may explain differences in post-exercise cTn-levels between individuals (Shave et al., 2010). We tested this hypothesis by using linear regression analyses, but were unable to find any significant associations (*P =* 0.12). However, it is likely that a higher absolute intensity would have resulted in an increased release of hs-cTnT; the average speed was quite low at 9.0 km h^−1^. It is also important to consider the altitude of 2,400 m in which the 78 km race was performed. Even though all the participants were born and grew up under similar moderate high-altitude conditions, the low oxygen partial pressure most likely affected the absolute intensity of the race, and thus the general strain on the cardiac muscle.

Myocardial stress in the form of wall-stretch caused by volume and pressure overload may be evaluated using natriuretic peptides as a proxy (Wu and Smith, 2004) such as the MR-proANP, secondarily using LDH, which primarily mirrors exercise-induced skeletal muscle damage (Lippi et al., 2006) even though it has also been used to determine cardiac necrosis (Gaze and Collinson, 2005). Unexpectedly, we found very quick clearance of mean plasma MR-proANP, i.e. by 1 h post-exercise, and mean plasma LDH was not significantly different from baseline at any time post-exercise. As already discussed, it is likely that the comparatively low average absolute intensity of the race, despite taking place at altitude with less available oxygen, did not put enough strain on the myocardium to increase the levels of the cardiac plasma markers.

The mean copeptin-us plasma concentration was elevated compared to baseline up to 3 h post-exercise, which may reflect chronic percentage of plasma volume vasopressin secretion under conditions of endurance exercise (Hew-Butler et al., 2011). This is explained by an up-regulation of the arginine-vasopressin system during a stress-response (Lin et al., 2012). Copeptin-us is also a marker of renal function, and the post-exercise increase in copeptin-us concentration may be due to exercise-associated systemic plasma volume depletion, i.e. dehydration which results in weight loss and an increase in plasma osmolality (Szinnai et al., 2007); unfortunately this was not measured in the present study. Furthermore, we measured mean post-race plasma creatinine as a proxy of acute kidney damage and/or dehydration showing a 25% increase at the 6 h time-point. We cannot fully explain the delayed plasma increase of creatinine, and the lack of an immediate post-exercise increase – even though borderline significant (*P =* 0.075)—is in contrast to results from previous studies in runners completing a marathon race (McCullough et al., 2011; Trivax et al., 2010) or to runners completing a 100 km race (Kao et al., in press). The rise in plasma creatinine may also be due to skeletal muscle activity, but it is equally possible that the physiological stress—despite the low average speed—does reflect a decrease in renal filtration function and not merely skeletal muscle damage.

Several of the plasma markers measured in this report may be indicators of foot-strike hemolysis, even though conflicting results have been reported on the burden of foot-strike hemolysis as a result of long-distance running, some showing a relationship (Lippi et al., 2012a) while others failed to show a relationship (Robinson et al., 2006). Plasma haptoglobin, which binds free hemoglobin, decreased significantly up to 24 h post-exercise, even though all mean values remained within the normal lower range of 0.6 g/L. Thus, even though intravascular hemolysis did occur, chronic “sports anemia” was probably not a factor, despite the fact that two of the participants had mild anemia at baseline. Anemia in the Tarahumara could be a consequence of inadequate nutrition which is not uncommon (Monarrez-Espino et al., 2001). A statistically significant correlation between total CK and LDH indicates that the latter is primarily related to skeletal muscle damage in spite of a lack of post-exercise increase in plasma LDH. Furthermore, plasma iron decreased at 3 h post-exercise compared to baseline. A delayed post-exercise plasma iron decrease has previously been shown (Robson-Ansley et al., 2011), and in a review on the compromise of an athlete's iron status, Peeling and co-workers (Peeling et al., 2008) emphasized hemolysis, hematuria, gastrointestinal bleeding and sweating as the most important explanatory mechanisms.

Finally, we also measured hs-CRP which is the most commonly used inflammatory plasma marker. Interestingly, we found mean hs-CRP of ∼16 mg/L (range 11–25 mg/L) at baseline, which has been associated with increased risk (>3 mg/L) of future cardiovascular disease (Ridker et al., 2002). However, it is likely that the apparently chronically high CRP concentrations are due to underlying infections which are not uncommon in rural, indigenous populations (Khambalia et al., 2011). The mean acute phase response of hs-CRP which we observed increased up to 24 h by 150% but had returned to baseline level by 48 h. Similar relative increases in CRP and return to baseline level 48 h post exercise of endurance sports has previously been reported (Taylor et al., 1987).

It is of note that the estimated mean VO_2max_ of 48 mL0_2_ min^−1^ kg^−1^, although not directly measured with respiratory gas analysis during maximum exercise test, is not very high for runners capable of completing an ultra-distance race of 78 km. In comparison, European amateur runners participating in a 24 h ultra-marathon race—an event not unfamiliar to many Tarahumara runners—and with a slightly older mean age had mean VO_2max_ of ∼58 mL0_2_ min^−1^ kg^−1^ (Waskiewicz et al., 2012). In the current study, certainly not all the participants were trained distance runners which is indicated in the range of estimated VO_2max_ of 34 to 61 mL0_2_ min^−1^ kg^−1^ as well as the range of running speed of 7 to 12 km h^−1^. The slowest of the runners were walking a considerable part of the 78 km distance. Previous studies in the Tarahumara have shown a similar range in estimated VO_2max_ (Aghemo et al., 1971) and average running speed for a similar distance (Groom, 1971). Nevertheless, relative intensity (% VO_2max_) was high with a mean of 68% (range 61–79%), and together with five out of six subjects having moderate or mild hypoxemia up to 30 min post-race, this indicates that the runners were able to push themselves considerably physically as well as mentally. However, despite marked acute-responses to the 78 km running race, all cardiac, renal and hemolysis plasma markers were back to baseline 48 h after the race. Thus, exercise-induced cardiac damage, kidney damage, and hemolysis in the Mexican Tarahumara are transient, which may be due to a combination of the low absolute intensity of this 78 km race and/or life-long adaptation to long-distance running at low absolute intensity.

We acknowledge several limitations to this observational study. First of all, the lack of a control group makes it impossible to make direct comparisons with other runners for an assessment of ethnic differences on exercise-induced cardiac or renal damage as well as hemolysis. Thus, this study should be seen as a baseline report on resting and exercise-induced key plasma markers in the Tarahumara. Another limitation is the lack of measurements on hydration during and after the 78 km race, even though we do have indirect measurement of dehydration with the measurements of plasma creatinine and copeptin-us. Furthermore, we only used indirect methodology to determine VO_2max_ which may result in a ±10–15% deviation compared to an indirect calorimetry measurement. Finally, we did not analyze the conventionally used brain natriuretic peptide or N-terminal brain natriuretic peptide for the assessment of wall-stretch caused by volume and pressure overload, but instead the novel MR-proANP, which limits the comparability with other studies.

### Perspectives

This study combines ultra-distance running and athletes familiar with such extreme physical exertion with known and novel plasma markers for cardiac and kidney damage as well as hemolysis, and thus has the potential to be an experimental model for acute physiological strain and time to full recovery in athletes. The results from the current study suggest that athletes engaging in ultra-distance running need to partly or fully recover for up to 48 h following exercise of ∼7 h or more before resuming training/competition. Intensity in combination with distance of running should be the focus of future research when investigating the degree of exercise-induced damage of key organs such as the heart and the kidneys in athletes. This focus should go hand-in-hand with measurements of genetic susceptibility to exercise-induced plasma marker release to unravel gene-environmental interaction.
